# Supplementary material for: External Validation of Deep Learning Models for Classifying Etiology of Retinal Hemorrhage Using Diverse Fundus Photography Datasets
Source: Bioengineering (Basel). 2024 Dec 29;12(1):20. doi: 10.3390/bioengineering12010020 (PMC11760437; doi:10.3390/bioengineering12010020)
Supplement: Supplementary file 1 [file bioengineering-12-00020-s001.zip › bioengineering-3331153-supplementary.pdf]

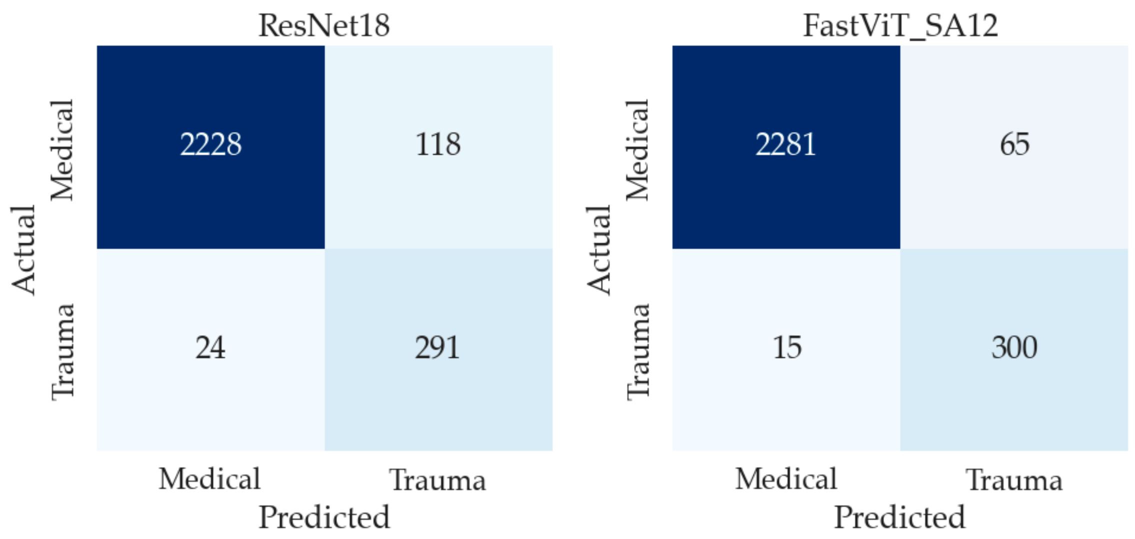

Figure S1. Confusion matrices for ResNet18 **(left)** and FastViT-SA12 **(right)** models on the external validation dataset. Each matrix compares the models' predictions against actual labels for medical and trauma etiologies of retinal hemorrhage.

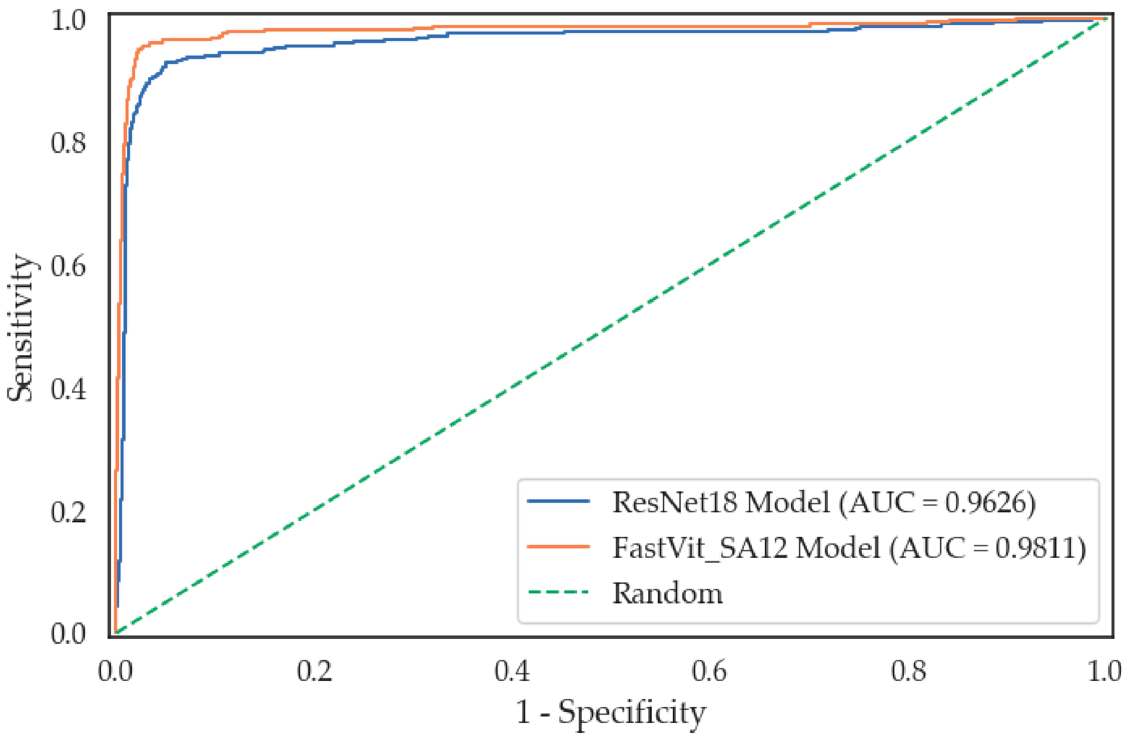

Figure S2. Receiver Operating Characteristic (ROC) curves for ResNet18 and FastViT-SA12 models on the combined private and public external datasets. The ROC curves illustrate the trade-off between sensitivity and specificity for both models. FastViT-SA12 achieved a higher area under the curve (AUC = 0.9811), indicating superior overall performance, while ResNet18 achieved an AUC of 0.9626. The green dashed line represents a random classifier for comparison.
